# Accessing Mental Health Services: a Systematic Review and Meta-ethnography of the Experiences of South Asian Service Users in the UK

**DOI:** 10.1007/s40615-021-00993-x

**Published:** 2021-03-08

**Authors:** Riddhi Prajapati, Helen Liebling

**Affiliations:** Faculty of Health and Life Sciences, Coventry University, Priory Street, Coventry, CV1 5FB UK

**Keywords:** South Asian, Help-seeking, Mental health services, Healthcare utilisation, Meta-ethnography, Qualitative research

## Abstract

**Background:**

Despite calls to address ethnic inequalities to accessing mental health services in the UK, governmental initiatives have had limited impact. Studies indicate that South Asian communities underutilise mental health services. Previous reviews have identified cultural and institutional factors that may influence service use, but these are mostly narrative and limited in their scope.

**Method:**

A systematic literature search resulted in fifteen studies exploring the experiences of seeking help and barriers to accessing and using services from the perspective of British South Asian service users.

**Findings:**

Qualitative data was synthesised through meta-ethnography, and three themes emerged: *Distanced from Services*, *Dilemma of Trust* and *Threat to Cultural Identity*. South Asian service users were positioned at a distance from being able to access services and stuck in a dilemma of mistrusting White and Asian professionals. They constructed their cultural identity through a set of important values which were neglected by mental health services. Service users, therefore, appeared to engage in an ongoing evaluation of the potential benefits of accessing services against the risks of threat to their personal and cultural identities. The findings are discussed in relation to Eurocentric models of care and community engagement approaches.

**Conclusion:**

The review argues that institutional racism and cultural dissonance marginalise South Asian service users from access to quality and effective mental healthcare. It is recommended that services acknowledge the impact of alienation and powerlessness and advance their practices to establish trust and cultural safety for South Asian service users in the UK.

## Introduction

### Political Context

Ethnic inequalities have been rising over recent decades in UK mental health services, leading to government initiatives to identify barriers and address disparities in outcomes and experience for Black, Asian and Minority Ethnic (BAME) groups. This includes the policy documents Inside Outside [1] and Delivering Race Equality in Mental Health Care [2]. The latter action plan aimed to achieve equality by pledging that “everyone who experiences mental ill-health is entitled to a safe and clinically effective, recovery-enhancing environment that respects their beliefs, culture, faith, spiritual needs and values” [2].

Despite significant investment, outcomes from these programmes have failed to achieve lasting impact in areas of policy and service provision [3]. Findings confirm little improvement in key measures of race equality, with worsening outcomes for some service users [4]. This has been ascribed to the culture of the statutory sector which appears stuck and “impervious to much change” [5]. However, failure to meet the needs of BAME groups has been argued to constitute institutional racism, since it places service users at a disadvantage in terms of attaining good health and standard of living compared to the majority White population [6].

### Mental Health of South Asian Communities

The ethnic category of “South Asian” refers to people whose familial or cultural backgrounds originate from countries on the Indian subcontinent, including India, Pakistan, Bangladesh, Nepal, Bhutan and Sri Lanka. These communities are markedly heterogeneous given the huge religious, linguistic, cultural and economic diversity among and between ethnic groups originating from these countries. Although South Asian migrants have been settling in the UK since the seventeenth century [7], the current populations are largely the result of immigration following the Second World War from former British colonies or Commonwealth countries, encouraged by British colonial authorities due to postwar labour shortages [8]. According to the last UK census in 2011, 7.5% of the total population identified as “South Asian”, comprising the largest ethnic minority in the UK [9]. Ongoing immigration since then means that India and Pakistan were among the top three most common countries of origin for UK migrants in 2019 [10].

Research points to social determinants as important considerations for mental health outcomes in South Asian groups. Racism and discrimination, both direct and indirect, have been common experiences for these communities since they began settling in the UK [11, 12]. British South Asians are also adversely impacted by structural socioeconomic disadvantages such as poor educational opportunities, inadequate housing, poverty and unemployment [13, 14]. Combined, these experiences are known to negatively impact psychological wellbeing through maintaining material deprivation, social exclusion and intergenerational trauma [12]. Growing evidence supports that South Asian people have higher levels of psychological distress compared to the White majority in the UK [15, 16].

### Help-seeking and Access to Mental Health Services

Despite widespread social and psychological difficulties affecting the mental health of British South Asian communities, it has been established that they utilise mental health services less than other ethnic groups [17]. This underutilisation has commonly been ascribed to differences in help-seeking behaviours [18]. Help-seeking can be defined as “attempts to maximise wellness or to ameliorate, mitigate, or eliminate distress” [19]. Since culture is one of the largest determinants of behaviour across ethnic groups, culturally informed views naturally influence understandings of distress, help-seeking and healthcare utilisation [20].

Previous reviews have identified cultural factors that may influence help-seeking for South Asian groups. These include stigma [21], collectivist values and pluralistic treatment options [22], as well as religious beliefs and somatisation [15]. Although these findings highlight the role of individual differences and cultural “explanatory models” in help-seeking, they neglect the roles of systemic sociopolitical factors in causing and contributing to distress and healthcare systems in perpetuating underutilisation through their ongoing failures to address institutional racism and ethnic inequalities. For example, compared to other ethnic groups, general practitioners (GPs) are less likely to recognise mental health difficulties in South Asian groups [23] and are less likely to refer them to specialist services even after recognition [24]. South Asian service users are also less likely to be referred for talking therapies [25], despite some reporting a preference for this over psychiatric medication [26].

Access to effective mental healthcare can, therefore, be conceptualised through a model of “candidacy”, where eligibility for professional attention and intervention is jointly negotiated between individuals and healthcare services [27]. Services can be constructed through a spectrum of permeability, where “high” permeability services are easily accessed and negotiated, whereas “low” permeability services demand more work, are more resistant and present more barriers. It has been highlighted that South Asian groups are at risk in their attempts to assert their candidacy for mental healthcare due to socioeconomic, cultural and institutional exclusion from statutory services [28, 29], where cultural exclusion is the inability to provide appropriate understanding for service users who do not belong to the majority White population and institutional exclusion is the inability to acknowledge specific needs, such as those related to ethnicity.

Part of these failures can be attributed to the Eurocentricity of mental health services in the UK, whereby the norms, values and practices of Western culture are privileged over and above those of non-dominant cultures [5]. This can be seen in the “colour-blind” or “one-size-fits-all” models and practices adopted by healthcare professionals in various disciplines. Psychiatric diagnoses are known to oversimplify and obscure significant and complex sociocultural processes such as racial trauma and socioeconomic inequality, leading to reductionist and medicalised approaches to understanding emotional distress that cannot accurately represent the lived experiences of all groups [30–32]. Psychological theories and models are also known to disproportionately represent the experiences of WEIRD (Western, Educated, Industrialized, Rich, Democratic) populations [33], which undermines their explanatory power but maintains methods of therapeutic assessment and intervention that are grounded in individualism. These universally applied Eurocentric assumptions marginalise and “other” the shared norms, values and practices of South Asian groups, such that their attempts to seek help from statutory services are consciously or unconsciously stereotyped and pathologised by healthcare professionals [25, 34, 35], often resulting in them being labelled as “hard to reach” [36].

### Rationale for the Current Research

Research exploring ethnic inequalities in accessing mental healthcare has predominantly used epidemiological approaches; although this highlights different pathways into services, it provides no depth of understanding from service users themselves [28]. It has been recommended that primary care trusts and local authorities engage with BAME communities to ensure that “care and recovery planning processes include service users’ perspectives of their needs” [2]. However, the views and experiences of South Asian service users have been a neglected area in qualitative research to date; where their views have been represented, they are often amalgamated with other ethnic groups or with professionals and service providers, undermining the importance of their specific views.

Furthermore, existing literature on the views of South Asian service users has focused on specific psychiatric diagnoses or inpatient settings. Since GPs are among the most accessible and “permeable” service for these groups [27] and 90% of people with mental health difficulties are treated in primary care [2], research also needs to focus on community settings [37, 38]. Therefore, this review aimed to fill a gap in the literature by synthesising qualitative evidence on the experiences of British South Asian adults when accessing community mental health services. It had two primary questions: What experiences do South Asian service users have of accessing mental health services in the UK? What do South Asian service users perceive as barriers to accessing mental health services in the UK?

The method of meta-ethnography was employed to identify and systematically synthesise the qualitative evidence. It was hoped that this would enable a deeper understanding of the experiences of South Asian service users, in order that mental health services make lasting improvements to address the identified barriers and better meet their needs [39]. The ENTREQ (enhancing transparency in reporting the synthesis of qualitative research) statement was used to guide reporting of the methodological process carried out in the review [40]. This allowed for clear and comprehensive reporting of important stages in the review according to evidence-based standards, including the systematic literature search, quality appraisal of studies and qualitative synthesis of the findings.

## Method

### Systematic Literature Search

#### Search Strategy

A comprehensive preplanned literature search was carried out to identify relevant primary research studies conducted in the UK from 1999 to 2019. This time period was chosen because the National Service Framework for Mental Health [41] set out key targets for improving access to mental health services in 1999, highlighting that mental health difficulties were frequently overlooked in BAME communities and that people from BAME communities were less likely to be referred to psychological therapies. This coincided with the Race Relations (Amendment) Act 2000, which made it unlawful to discriminate on the basis of race, ethnicity or culture and placed a duty of care on public services such as the National Health Service (NHS) to target racial discrimination and actively promote equality.

The PICOS framework was used to structure the search across research databases (Table 1). This framework is a modified version of the popular PICO tool, which focuses on the Population, Intervention, Comparison and Outcome of an article, with additional terms for the study design to limit the number of irrelevant articles. The PICOS tool has shown to be effective for reviews of qualitative studies [42]. Additional terms for geographical region were included to filter studies conducted within the UK.

**Table 1:** Search terms applied in the systematic literature search

	Concept	Synonyms	Location
Population	South Asian ethnicity	South Asian, Asian, Bangladeshi, Bengali, Gujarati, Indian, Sri Lankan, Pakistani, Punjabi	Title Abstract Keywords Subject headings
Intervention	Mental health and/or mental health services	Mental health, mental illness, mental disorder, emotional distress, psychological distress, mental health services, psychological services, primary care, community mental health services, psychotherapy, therapy, counselling	Title Abstract Keywords Subject headings
Comparison	N/A		
Outcome	Help-seeking and barriers to service use	Acceptability, access, accessibility, barriers, challenges, engagement, exclusion, hindrance, limitations, obstacles, pathway, use, utilisation, help-seeking, seeking support	Title Abstract Keywords Subject headings
Study type	Qualitative	Qualitative, interview, focus group, content analysis, discourse analysis, ethnography, grounded theory, mixed methods, narrative, phenomenological, thematic analysis	Title Abstract Keywords Subject headings
Geographical region	UK	England, Scotland, Wales, Northern Ireland, UK, Great Britain	Title Abstract Keywords Subject headings

#### Search Outcome

**Fig. 1:**
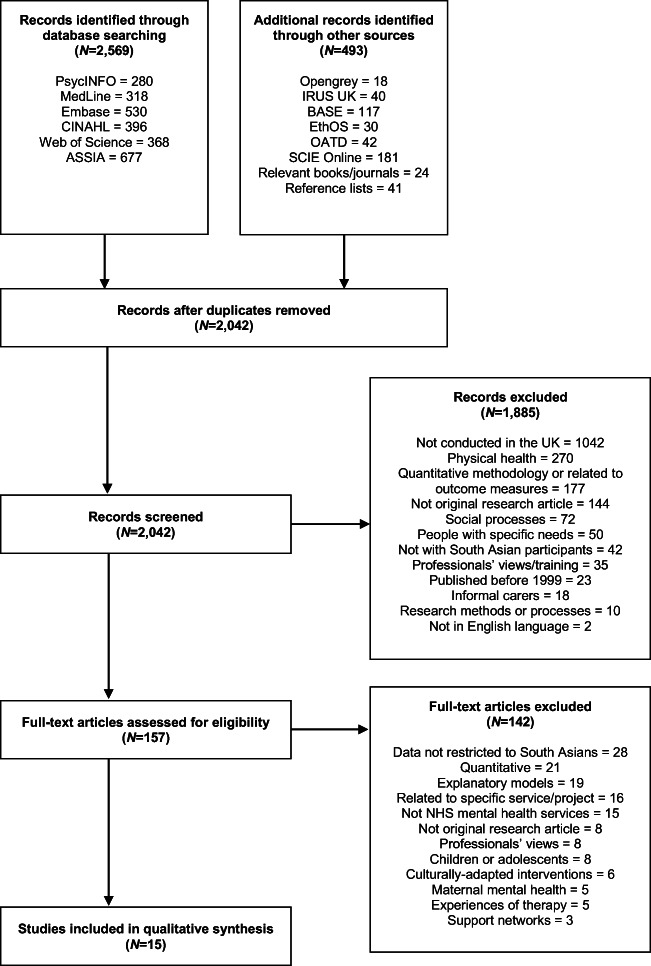
PRISMA flow diagram

A PRISMA flow diagram was used to illustrate the study selection process [43]. Figure 1 reveals the electronic research databases and relevant grey literature searched. In line with previous recommendations for conducting a meta-ethnography, additional articles were identified by searching through the citations of selected articles and literature reviews, reviewing references lists and hand-searching relevant books and journals [39, 44]. All records were imported into RefWorks reference management software for management. After eliminating duplicates, the titles and abstracts of 2,042 records were manually screened against the inclusion and exclusion criteria by the lead researcher (Table 2). Where this was not possible due to insufficient information in the title and abstract, full-text articles were obtained and assessed against the criteria. An inclusive approach was employed to avoid omitting research of potential value [44].

**Table 2:** Inclusion and exclusion criteria for study selection

	Inclusion criteria	Exclusion criteria
**Geographical region**	Conducted in the UK	Conducted in any country other than the UK
**Time period**	Published between 1999 and 2019	Published before 1999
**Methodology**	Qualitative methodology for data collection and analysis	Quantitative methods
**Population**	South Asian participants (Indian, Pakistani, Bangladeshi) whose data was analysed separately	Studies which combined data of South Asian participants with participants from other ethnic groups
	Potential or actual service users	Family members, healthcare professionals or community leaders
**Age**	Adults (aged over 18 years)	Children or adolescents
**Concepts**	Experiences of seeking help in statutory mental health services	Not relevant to issues of access or use of statutory mental health services
	Barriers to accessing statutory mental health services	Focused on a specific service or intervention
**Article**	Original research article	Not an original research article, e.g. review, report, book chapter
**Language**	Article written in English	Article written in language other than English

This resulted in fifteen relevant studies which satisfied the inclusion criteria and were retained for quality assessment. Given that the aim was to provide a depth of insight into the help-seeking experiences and barriers perceived by South Asian service users, the number of included studies allowed for a rich description of experiences and conceptual clarity.

### Quality Appraisal

The quality assessment tool devised by the Critical Appraisal Skills Programme (CASP) was used to appraise the quality of the fifteen identified studies [45]. This structured quality appraisal tool considers key methodological components relevant to qualitative research using ten criteria. The CASP tool was chosen as it has been developed and tested over a period of time [46], has been used by several researchers to appraise the quality of research studies for meta-ethnography [44, 47] and has shown to be effective in achieving this purpose [48].

Each study was assessed against ten criteria to determine the validity and utility of its findings. A rating system was used whereby 0 = *criterion not met*, 1 = *criterion partially met* and 2 = *criterion fully met* [49]. Similar to the practice of other researchers [50], the quality of studies was assessed by the overall rating rather than employing a cutoff score, where *high quality* = 16–20, *medium quality* = 11–15 and *low quality* = 0–10. This allowed for all relevant studies to be included in the review.

The lead researcher and an independent reviewer rated each study separately. Inter-reliability was then assessed using Cohen’s kappa (*κ*) coefficient. Significant discrepancies between ratings were discussed to improve inter-rater reliability. The final reliability coefficients ranged from *κ*=.615 to κ=1 (Table 3), with an overall score of *κ*=.852, representing a moderate to strong pattern of inter-rater reliability. Quality assessment ratings (QARs) ranged from 15 to 20 indicating that studies were of medium to high quality.

**Table 3:** Characteristics of the identified studies

Author(s) Study aims	Sample demographics	Study methodology and characteristics	Main relevant findings	QAR and reliability coefficient (*κ*)
**Bhui, Chandran and Sathyamoorthy (2002)**				
To understand South Asian men’s perceptions of mental health assessment and to explore the quality of care available to male Asian service users	*N*=8 All male Age range 19–62 4 Indian, 2 Pakistani, 2 unclear 7 first-generation, 1 second-generation Conducted in South London	Actual service users Purposive sampling from statutory and voluntary mental health services Individual semi-structured interviews Analysed using thematic analysis Published article	Eight themes were found: (1) clinical contact, (2) professional role, (3) language and interpreters, (4) ethnicity and gender, (5) religion and culture, (6) understanding the problem, (7) reflection on assessment and (8) treatment Professionals often did not explain their role or the process of assessment. Participants spoke English to different levels but were assessed in English. They were not given a choice about the ethnicity/gender of professional, but some participants had clear preferences. Participants were not satisfied with the explanations given, had limited opportunity to discuss their perspective of the problem and were not satisfied with the treatments recommended. None were given a chance to discuss their religion/culture, yet all felt this would have been useful	QAR=17 *κ*=.756
**Bowl (2007)**				
To gain the specific views of South Asian service users about their interactions with mental health services and their perceived limitations	*N*=26 11 male, 15 female Age range 21–60+ Pakistani or Indian Generational status unclear Conducted in Birmingham	Actual service users Opportunistic sampling from statutory and voluntary mental health services Three focus groups (8 males, 7 females, 8 females, respectively) Notes analysed using thematic analysis Published article	Four themes were found: (1) socioeconomic exclusion, (2) cultural exclusion, (3) institutional exclusion and (4) wider views on services Many participants identified links between their mental health problems and socioeconomic exclusion, i.e. loss of employment and money. Many participants felt that their language needs were neglected, that assessments were not culturally relevant and that facilities were not provided for prayer, leading to cultural exclusion. They reported concerns that families were not involved in their care or that effort was not made to share knowledge about medications. Participants had strong support for resources targeted at South Asian service users and a view that commissioners were not doing enough to meet that need or engage them in decision-making	QAR=16 *κ*=.783
**Chew-Graham, Bashir, Chantler, Burman and Batsleer (2002)**				
To explore South Asian women’s perceptions and experiences of mental distress, attempted suicide/self-harm and barriers preventing access to services	*N*=31 All female Age range 17–50 Pakistani, Bangladeshi, Indian Generational status unclear Conducted in Manchester	Potential service users Purposive sampling from voluntary women’s groups in the community Four focus groups (with 5, 7, 7 and 12 participants, respectively) Analysed using framework analysis Published article	Ten themes were found: (1) izzat, (2) community grapevine, (3) racism, (4) English language problems, (5) psychological distress as a symptom of external pressures, (6) attempted suicide and self-harm as a response to social isolation, (7) domestic violence and consequences of leaving the family, (8) differences within communities, (9) access to mainstream service provision and (10) improving service provision. The importance of izzat (family honour and reputation) in Asian culture and family life was described, resulting in a need to protect izzat. A well-developed community grapevine often led to oppression and stigma from the community. Inability to speak English led to a lack of knowledge of services and support, whilst sexism and racism exacerbated the participants’ sense of isolation. All participants said trust was important to access mainstream services. Barriers included a lack of understanding of the Asian culture, providers being mostly White, issues with interpreters, fear of gossip, lack of awareness of services provided and little ethnic-matching	QAR=16 *κ*=.808
**Gilbert, Gilbert and Sanghera (2004)**				
To explore South Asian women’s views on shame, subordination and entrapment and how these might affect mental health problems, help-seeking and the use of services.	*N*=Not stated All female Age range 16–41+ Ethnicities not stated Generational status unclear Conducted in Derby	Potential service users Purposive sampling from a voluntary service Three focus groups of different ages (16–25, 26–40, 41+) in which four scenarios were presented with questions Method of analysis not stated Published article	Six categories were listed, four of which related to the four scenarios presented: (1) izzat scenario, (2) shame scenario, (3) subordination scenario, (4) entrapment scenario, (5) effects on mental health and (6) help-seeking behaviour Participants felt that help-seeking was influenced by fear of discovery, confidentiality and feeling ashamed. Participants did not know what services were available and were undecided on the benefits of seeing a White professional due to fears of being misunderstood	QAR=17 *κ*=.778
**Hussain and Cochrane (2002)**				
To explore the thoughts, feelings and beliefs of South Asian women on causes and cures for their depression and the implications for mental health services	*N*=13 All female Age range and ethnicities not stated 9 first-generation, 4 second-generation Conducted in Birmingham	Actual service users Non-probabilistic sampling from statutory mental health services Individual semi-structured interviews Analysed using grounded theory Published article	Three categories were identified: (1) conflicting cultural expectations; (2) distinctions between psychosocial, spiritual and physical problems; and (3) communication problems (general and culture specific) Participants reported that communication problems led to a lack of information, affected professionals’ interpretation of their problems and resulted in a lack of opportunity to access counselling/psychotherapy. They described minimal distinctions between psychosocial, spiritual and physical problems, leading to confusion over cause and subsequent treatment options, but also conflict with the restricted perspectives of mental health services	QAR=18 *κ*=.706
**Hussain and Cochrane (2003)**				
To explore beliefs and attitudes around coping and the strategies employed by South Asian women and the relationship between coping and treatment	*N*=13 All female Age range and ethnicities not stated 9 first-generation, 4 second-generation Conducted in Birmingham	Actual service users Non-probabilistic sampling from statutory mental health services Individual semi-structured interviews Analysed using grounded theory Published article	One core category was found: coping strategies used and factors affecting choice Coping strategies included praying/religion, herbal remedies, talking, self-harm or crying. Factors affecting the degree of coping were the perception of the problem, existence of motivating factors and access to help. Some participants felt they did not need to look externally for support and could cope by themselves, whereas others felt that external support was not available to them. Where support was identified, its use depended on weighing up the benefits of receiving community help against the problems this may cause	QAR=17 *κ*=.706
**Hussain (2006)**				
To understand the perceptions and experiences of Punjabi immigrants on accessing mental health services	*N*=33 16 male, 17 female Age range 55–62 All Punjabi Generational status unclear Conducted in North England	Actual service users Purposive sampling from statutory mental health services or traditional healers Individual semi-structured interviews Analysed using grounded theory Published article	Eight categories were identified: (1) a comprehensive understanding of the narratives of health and the experience of service use, (2) Punjabis’ beliefs regarding distress, (3) distress: causes and remedies, (4) kismet: fate as the cause of distress, (5) sabr: endurance of distress as help-seeking, (6) purdah: gender modesty and role fulfilment as the determinant activity in distress and its amelioration, (7) izzat: honour and family protection as the measure of balanced mental health and (8) “peace of mind”, normal mental health and its cultural formulation The participants’ understanding of distress was captured by their cultural and religious values (kismet, sabr, izzat and purdah), which were incongruent with Western concepts. Their use and experience of statutory services were filtered through the lens of these values, creating expectations of the means/content of service delivery that were incompatible with mainstream services. This led to confusion, alienation or further distress for both the participant and the provider, creating barriers to appropriate care	QAR=19 *κ*=1
**Kai and Hedges (1999)**				
To explore Pakistani and Bangladeshi service users’ perceptions of distress and its amelioration, in order to develop services appropriate to their needs	*N*=104 38 male, 66 female Age range 16–65+ 49 Pakistani, 55 Bangladeshi Generational status unclear Conducted in Newcastle	Potential service users Snowball sampling from community networks Individual semi-structured interviews facilitated by trained community project workers Method of analysis not stated Published article	Eight categories were listed: (1) racism, social disadvantage and distress, (2) coping with distress, (3) family relationships, (4) seeking help from professionals, (5) younger people, (6) community project workers’ experiences, (7) experiences of project steering group and costs and (8) subsequent service development. Most participants located their main sources of distress within their external social environment, related to racism, social disadvantage and distress. Some also raised their family and marital relationships as causes of distress, worry or sadness. A majority of participants felt that health professionals and GPs were unable to deal with worry or stress, defining their roles in terms of physical health. Most wanted to seek help from those who they felt would have understanding and empathy due to a shared background/culture, but they emphasised breach of confidentiality as a potential barrier	QAR=18 *κ*=1
**Mahmood (2012)**				
To explore the experiences and needs of Punjabi Pakistani immigrants with psychological distress and their views on using mental health services	*N*=7 All male Age range 21–35 Punjabi Pakistani All first-generation Conducted in London	Potential service users Convenience and snowball sampling from community centres Individual semi-structured interviews Analysed using IPA Unpublished doctoral thesis	Two superordinate themes were found: (1) on being masculine and (2) the unknown territory of counselling Participants described avoidance of confronting a weaker/vulnerable part of themselves, through self-reliance, presenting a strong image and restricting emotional expression. They also emphasised their lack of awareness of counselling services. Shame and stigma were attached to people struggling with their mental health, so participants had no strong motivations to access psychological services, instead, seeing therapy as a last resort	QAR=19 *κ*=1
**Moller, Burgess and Jogiyat (2016)**				
To explore barriers to counselling and help-seeking in second-generation South Asian women	*N*=82 All female Age range 18–40 34 Indian, 20 Pakistani, 10 mixed/other All second-generation Conducted in North England	Potential service users Opportunity sampling from community centres Open-ended questionnaires Analysed using thematic analysis Published article	One overarching theme of “stereotyping” was found, with four subordinate themes: (1) White counsellors are…, (2) Asian counsellors are…, (3) counselling is… and (4) people with psychological problems are… Participants held stereotypes about White and Asian counsellors which affected their choices to seek help. They saw White counsellors as culturally ignorant, yet nonjudgmental, and Asian counsellors as untrustworthy, yet understanding of cultural issues. Their choice depended on the nature of the problem they were hoping to seek help for. Participants saw counselling as an “abnormal” practice which brought shame and stigma to the family, as people with psychological problems were themselves stigmatised as “mad”	QAR=18 *κ*=.615
**Patel (2016)**				
To explore how the Indian Gujarati community understand mental health and make sense of help-seeking for mental health problems.	*N*=9 4 male, 5 female Age range 24–65 All Indian Gujarati 4 first-generation, 5 second-generation Conducted in London	Potential service users Opportunistic and snowball sampling from community centres and temples Individual semi-structured interviews Analysed using thematic analysis Unpublished doctoral thesis	Five themes were found: (1) constructions and causes of mental health problems, (2) religion: an integral role, (3) community: a means of support and safety, (4) family: honour and reputation and (5) professional services: challenges and vision Participants perceived religion as integral to their daily life and a vital coping resource/protective factor. Family were often the first source of support for participants; they talked about the significance of protecting family honour by not sharing problems with others. The community was positioned as having a unique role in providing support, which participants saw as being central in seeking help for mental health difficulties. A number of barriers to help-seeking were reported, including a lack of cultural sensitivity, language issues, fear of gossip, damage to marriage prospects and a lack of trusting relationship with professionals. Many felt that services needed to make links with community/faith groups to overcome these barriers	QAR=18 *κ*=1
**Ruprai (2016)**				
To explore beliefs about psychological wellbeing and an understanding of mental health issues in the Punjabi Sikh community	*N*=8 4 male, 4 female Age range 28–70 All Punjabi 8 first-generation, 3 second-generation Conducted in West London	Potential service users Opportunistic and snowball sampling from community centres and temples Individual semi-structured interviews Analysed using thematic analysis Unpublished doctoral thesis	Three themes were found: (1) we are warriors!, (2) the importance of family expectations and (3) understanding mental health issues Participants were strongly influenced by their Sikh history and believed that they were capable of coping with hardships without the input of external services. They believed that families should support each other through times of misfortune and that they do not suffer from “ill mental health” so did not see mental health services as relevant to them.	QAR=19 *κ*=1
**Sudan (2004)**				
To explore how South Asian men perceive their culture within a mental health context and to explore their perspective of having a psychiatric diagnosis and its implications	*N*=6 All male Age range 30–40 Pakistani, Indian All second-generation Conducted in Leicester	Actual service users Purposive sampling from statutory mental health services Individual semi-structured interview Analysed using grounded theory Unpublished doctoral thesis	One overarching category of “reconstructing a sense of identity” was found, with five main themes: (1) identification with Asian culture and values, (2) contact and experience of the mental health system, (3) identity not being heard, (4) others influencing the formation of separate identities and (5) others influencing the integration of identities Participants felt that their Asian identity was not being heard by statutory services and questioned dominant beliefs about the origins and consequences of illness. Other people, including family and statutory services, were important in influencing the formation or integration of their identities	QAR=19 *κ*=1
**Thompson (2010)**				
To discover what second-generation Asians of Sikh faith require from older adult psychological services to promote their psychological health and wellbeing	*N*=73 31 males, 42 females Age range 45–65 All Asians of Sikh faith Generational status unclear Conducted in Sandwell	Actual service users Purposive and snowball sampling from statutory mental health services and community organisations Individual semi-structured interviews Analysed using IPA Unpublished doctoral thesis	Eight main themes were found: (1) cultural and contextual background, (2) the significance of religion in health and healthcare, (3) individual strategies for managing distress, (4) individual strategies for enhancing quality of life, (5) challenges to quality of life in old age, (6) limited service provision, (7) all psychological services are potentially useful for this generation/community and (8) service delivery considerations for the Sikh community Good health was seen as a shared responsibility between God and the person. Religious coping strategies such as prayer and meditation were significant, and participants kept busy and active through their roles in the family and community. Participants wanted services to account for religious beliefs, showing persistent demonstrations of interest and concern to help them feel valued and looked after. Barriers to service use included fear of gossip and breaching confidentiality. Aids to support seeking included publicity, familiarity and encouragement	QAR=19 *κ*=1
**Wood (2011)**				
To explore the experiences and meanings of South Asian women who self-harm and their experiences and perceptions of support services	*N*=5 All female Age range 20–35 4 Pakistani, 1 Bangladeshi All second-generation Conducted in Leeds	Actual service users Purposive sampling from statutory mental health services and community groups Individual semi-structured interviews Analysed using IPA Unpublished doctoral thesis	Participants’ accounts were analysed individually, but three themes were identified at the group-level: (1) control, (2) communication and (3) identity Participants felt unable to express themselves due to feeling controlled by others and experienced conflict regarding their sense of self. They expressed fear of judgment as a barrier to sharing their distress; this was related to potential responses to their self-harm and the ethnicity of professionals. Service responses sometimes inadvertently replicated the patterns in their previous interactions and consequently exacerbated their distress	QAR=19 *κ*=1

*QAR*, quality assessment rating as rated according to the Critical Appraisal Skills Programme quality appraisal tool [45]

### Characteristics of the Literature

Studies varied in the information they provided regarding sample demographics. Several studies did not report the participants’ ethnicity or generational status, and one study did not report the sample size [51]. Two studies were conducted using the same sample so were only counted once, although both contributed to the analysis as they covered different topics [26, 52].

Based on the data provided, the following demographics were identified (Table 3). Sample sizes ranged from 5 to 104 participants. Service users ranged from 16 to 70 years in age and consisted of 280 females and 125 males in total. Participants identified their ethnicity as Indian, Pakistani or Bangladeshi and reported religious or faith traditions as Islam, Hinduism or Sikhism. Whilst three studies focused on second-generation adults [53, Sudan, 2004, Wood, 2011], most included a combination of first- and second-generation South Asian adults. Given the limited number of studies, it was not possible to use migrant or generational status as an inclusion/exclusion criterion. Instead, children and adolescents were excluded based on age.

Eight studies included clinical samples with experiences of statutory services in the community, whilst seven studies explored the perspectives of South Asian people with no prior experiences of seeking professional help. The attitudes and beliefs of “lay people” may not correspond with their actual intent or behaviour but offered the perspectives of those who may go on to experience emotional distress and seek support from services. Clinical samples reported experiencing depression, anxiety, self-harm, schizophrenia and psychosis. In three studies, participants had been in contact with community and inpatient services [28, 54, Sudan, 2004]; where possible, only their experiences relevant to community settings were included. For ease of reading, both potential and actual service users are referred to as “service user” in this review.

Studies predominantly employed individual semi-structured interviews to collect data. Three studies conducted focus groups [28, 51, 55], whilst one study used open-ended questionnaires [53]. These latter methods may have influenced the data collected since participants were not given the opportunity to explore their individual views. Furthermore, variable information was provided about the methodological process. Some studies gave minimal information about their analytic procedure, whilst others gave little consideration to researcher reflexivity. This made it difficult to appraise the rigour and validity of their qualitative analysis.

Nine studies were published journal articles, whilst six studies were unpublished doctoral theses identified in the grey literature. Although the unpublished doctoral theses were not peer-reviewed to the same extent as the published articles, overall they were of higher quality as indicated by their QARs.

The final fifteen studies represented the experiences of four hundred and five South Asian service users from different geographical regions of England. Key findings relevant to the aims of the review are shown in Table 3.

### Qualitative Synthesis

Meta-ethnography was chosen as the synthesis method for this review as it offers a means of rigorously synthesising qualitative research to provide a “range and depth of meanings, experiences, and perspectives of participants across healthcare contexts” [40]. It allows for a higher level of analysis compared to conventional literature reviews by enabling the construction of an interpretative layer which extends beyond the interpretations provided by the included studies [56]. Such a synthesis can achieve greater conceptual understanding and make valuable knowledge accessible to healthcare professionals, in line with the aims of this review [44]. To achieve this, the seven phases of meta-ethnography [57] were implemented in an iterative manner (Table 4).

**Table 4:** Seven phases of meta-ethnography applied in the qualitative synthesis

Phase	Description
**Getting started**	The initial focus of interest was on the help-seeking experiences of South Asian service users and barriers to accessing mental health services
**Confirming initial interest**	After scoping the literature and discussion with the research team, the initial interest was refined to studies in primary care or community settings and restricted to adults. A decision was made to include studies with non-clinical samples, as it was felt this could shed light on the barriers for “potential” service users
**Reading the studies and extracting data**	The findings section of the resulting fifteen studies were read and re-read with close attention to identify ideas or metaphors relevant to the review aims. These initial “concepts” were noted on the original studies by the lead researcher
**Determining how the studies are related**	Concepts were entered into a spreadsheet to enable comparison, whereby the concepts were entered into rows, and the identified studies were entered into columns in chronological order. The original list of concepts were lack of information/awareness, lack of collaborative care, preferences, mistrust of professionals, professionals not equipped, fear of breach of confidentiality, fear of being misjudged, fear of stigma, lack of cultural sensitivity, cultural differences, family privacy and honour, importance of language and importance of religion
**Translating the studies**	Using the spreadsheet, concepts were compared within and across studies by the lead researcher, referring to the original text where necessary, to develop concepts that represented “meaningful ideas that explain and not just describe the data” [58]. A process of “translation” enabled related concepts to be merged or revised, whilst others were reduced, giving rise to the final six concepts: *outside of awareness*, *outside of cultural norms*, *cannot trust White professionals*, *cannot trust Asian professionals*, *lack of collaborative care* and *lack of cultural sensitivity*
**Synthesising translations**	The six derived concepts were then further reviewed by the research team to establish the relationships between them; it appeared that they were not refutations of one another, but were reciprocal. This enabled a “line of argument” synthesis to be developed, which involved putting the concepts into interpretative order to make “a whole into something more than the parts alone imply” [57].
**Expressing the synthesis**	The six concepts were abstracted into three themes which comprised the final synthesis as follows: *distanced from services*, *dilemma of trust* and *threat to cultural identity* (Table 5).

## Findings

Following the meta-synthesis, three themes emerged: *Distanced from Services*, *Dilemma of Trust* and *Threat to Cultural Identity* (Table 5). The line of argument synthesis indicated that service users were positioned at a distance from being able to access services, either due to a lack of awareness of their availability and remit or due to cultural norms which discouraged them from seeking help outside the family. They were also stuck in a dilemma of mistrusting professionals from White and Asian ethnic backgrounds, due to legitimate fears of personal and social invalidation. Many service users experienced limited collaboration and negation of their cultural identity when accessing services, leading to feelings of powerlessness and alienation. This appeared to confirm their mistrust and reinforced their distanced position from services. They therefore engaged in an ongoing evaluation of the potential benefits of accessing services against the risks of threat to their personal and cultural identity.

**Table 5:** Themes and concepts

Themes	Concepts
**Distanced from Services**	Outside of Awareness
	Outside of Cultural Norms
**Dilemma of Trust**	Cannot Trust White Professionals
	Cannot Trust Asian Professionals
**Threat to Cultural Identity**	Lack of Collaborative Care
	Lack of Cultural Sensitivity

These themes and concepts are not necessarily sequential and may not apply to all South Asian service users. Rather, they represent the experiences reported by study participants and the barriers that appeared necessary to consider and negotiate when seeking help. Each theme is described together with the relevant concepts. Quotations are included from studies to illustrate the concepts.

### Theme 1: Distanced from Services

Service users were positioned at a distance from being able to access mental health services. This was either due to a lack of awareness of the availability or remit of services or as a result of cultural norms and practices which discouraged them from seeking help outside the family or community. The two concepts within this theme represent these barriers including *Outside of Awareness* and *Outside of Cultural Norms*.

#### Concept 1: *Outside of Awareness*

Eleven of the fifteen studies reported a lack of awareness or knowledge of mental health services as a barrier to seeking help. Service users were unaware of local community services and alternatives to biomedical treatment. Some questioned, “how do you find out about these services?” [59], whilst others relied on sources including the media for information. Many spoke about their confusion or uncertainty regarding the role of mental health professionals, illustrated by one participant in Mahmood’s study [2012]:

*Well, I don't know a lot about it. I know that the psychiatrist and the psychologist are the people who are dealing with those things I mean well broadly speaking, I don't have any idea of how these people work and I've never been in any organisation like that before.*

Approaching mental health services was often considered unnecessary. The family was viewed as the main source of support since there were strong intergenerational expectations about duty and responsibility to care for people with difficulties. The wider community was positioned as being protective of its members and assumed a collective responsibility to support one another in times of distress, as described by one male service user:

*If you have a network of people, who are saying to you ‘we understand that you have a problem, but we are going to do whatever it takes in order to help you’. That's when you know that you can get through it, because you're not alone anymore. That vulnerability kind of shifts, because you're not reliant on just yourself to get over it, and you know you have the support of everyone else*. [Patel, 2016]

Additionally, some service users viewed themselves as already having good psychological wellbeing, drawing on inner resources to cope with difficulties, including self-reliance and emotional control. This was attributed to values imbibed from family during childhood or from spiritual and religious teachings. Therefore when the use of services was suggested, some service users denied their applicability, saying “why I need because I know myself what I'm doing” [Mahmood, 2012].

#### Concept 2: *Outside of Cultural Norms*

When service users were aware of available services, studies showed that they were reluctant to seek help for fear of potential negative consequences. This included a fear of being labelled as someone with mental health difficulties, due to the “stigma attached to being a bit crazy or depressed” or “the impression of people who go to counselling” [53].

Studies reported that sensitive information was often privileged to the immediate family in Asian communities. Participants wondered “why would you want to wash your dirty laundry in public?” [Patel, 2016] and therefore did not share their concerns with others who were less well known and less trustworthy. Women stated that speaking openly about their feelings, especially in public, was frowned upon, whereas men were expected to uphold a strong demeanour and inhibit open displays of emotion, conforming to traditional constructs of masculinity. The notion of seeking help from a source outside the family was therefore regarded as not conforming to Asian cultural values and counter to family expectations.

Given the importance of the family unit, perceived shame and stigma naturally extended beyond the service user to their families and had the potential to impact on “izzat”, defined as family honour or reputation [51]. The consequences of this included damage to marriage prospects, negation of the family’s success, rejection or disownment and being shunned by the Asian community. In order to avoid this and protect izzat, mental health concerns were often confined within the family unit. This led to some service users feeling “trapped because she is bound by all the obligations of the family, to her responsibilities as a mother, as a wife, to society, to izzat, everything” [51].

### Theme 2: Dilemma of Trust

This theme represents the feelings of mistrust and fear that dominated service users’ perceptions and experiences of interacting with mental health services. Trust was reported as a fundamental requirement, since “people only seek help from people they trust” [Patel, 2016]. It contains two concepts: *Cannot Trust White Professionals* and *Cannot Trust Asian Professionals*.

#### Concept 1: *Cannot Trust White Professionals*

Across the reviewed studies, service users overwhelmingly reported a fear of being misjudged by White professionals. This was related to a view that White professionals were “culturally ignorant” and are “incapable of relating to a South Asian” [53]. Service users expressed concerns regarding how professionals would respond to them if they sought help. For example, judging them as an “Asian” woman, making naive statements which were actually racist or fixed on stereotypes or offering simplistic sweeping solutions like “leaving the family” without understanding the complexities of their experience. This “cultural dissonance” led to some service users feeling uncomfortable and disengaging from services.

Conversely, Asian professionals were perceived as sharing cultural understanding and values. This meant a better grasp of their difficulties and contextual situation, ease of sharing and talking and therefore a better understanding of them as an individual. Despite South Asian people coming from diverse ethnic, linguistic and religious backgrounds, one service user reported significant cultural similarities which enabled him to trust Asian professionals with information that “he would not share with others”, especially in settings where he was the minority [28].

#### Concept 2: *Cannot Trust Asian Professionals*

Despite Asian professionals potentially being more understanding of cultural issues, eight studies reported that service users were concerned about seeking help from them. These concerns were predominantly about confidentiality being breached, associated with a view that Asian professionals “gossip and judge regardless of what they may say to their bosses” [53]. Rumours were a threat to the family’s izzat since the Asian community was regarded as close-knit with a well-established “community grapevine” [55], in which “every movement we make is scrutinised” [Wood, 2011]. This meant that it would be impossible to have “full privacy when speaking to a counsellor” [53].

Examples were given of professionals informing the service user’s family of their concerns or disclosing their whereabouts after they fled domestic violence in the family. These experiences led to suspicions and huge mistrust of all statutory services, resulting in service users considering help-seeking as a last resort in desperate circumstances.

On the other hand, White professionals were perceived by South Asian service users as nonjudgmental and objective, as described by one service user:

*I would prefer a white person, because she is distant from the whole cultural situation, so she is more likely to be neutral*. [53]

This preference depended on the nature of the situation, as “if it was something that was stigmatised in my culture then I would definitely go to a white person” [53]. These mixed experiences led to a dilemma, whereby service users believed that they could not trust any professionals, Asian or otherwise. One service user expanded on this:

*You see no matter which GP you go to, a European one is going to come with his European values, and then he will try and be more objective but he will still be coming from that angle, and you know he will derive his cultural values from somewhere else, whereas an Asian GP will get them from somewhere else, so you are not going to get an objective point of view from anywhere really*. [51]

### Theme 3: Threat to Cultural Identity

Service users experienced help-seeking as bringing them into conflict with their needs, expectations and values, which negatively impacted on their sense of self. This related to their experiences as an individual seeking help at a time of distress and based on their cultural identity as a British South Asian adult. This theme contains two concepts: *Lack of Collaborative Care* and *Lack of Cultural Sensitivity*.

#### Concept 1: *Lack of Collaborative Care*

Service users described experiences of feeling disempowered and dehumanised when accessing services. Some reported that professionals would often not share pertinent information with them, such as their role, understanding of the difficulty, what to expect and the outcome of the assessment. GPs were seen to position themselves as experts, forcing service users and telling them what was required. Others were offered limited choices regarding their preferences, including gender or ethnicity of professional or treatment options, and felt that they were viewed as a diagnosis or a “statistic” [Ruprai, 2016]. One service user expressed dissatisfaction with this lack of collaborative care from professionals:

*They don’t sit you down and discuss the background and their thoughts on the illness and its causes and ask questions about different explanations—you know, all the things that help you arrive at an accurate diagnosis and help you work with the problem. It’s common knowledge that if two people present with similar symptoms, it doesn’t mean it’s the same illness…it’s about asking the right questions*. [28]

Furthermore, service users often valued the presence of family members for reassurance and support. One service user talked about the importance of involving the family in mental health care:

*It’s not an individualistic thing…within a South Asian culture…families don’t take decisions as individuals, you take major decisions together…the family would want to be included*. [Patel, 2016]

Despite this, service users felt that statutory services did not appreciate the importance of the family unit, ignored them regarding key decision-making processes and provided them with little support. This was deemed especially problematic for families who perceived themselves as having little knowledge or experience of mental health issues and those who were interested in providing support to the service user but were not given any opportunities to learn how to do this. Additionally, professionals rarely facilitated collaboration to improve the quality of care, instead involving family to mitigate the need for an interpreter.

#### Concept 2: *Lack of Cultural Sensitivity*

This concept highlights the dissonance between the cultural needs of South Asian service users and how these were met by services. Service users felt that professionals only asked about their “illness”, not the South Asian identity behind it. Salient information was not recognised, including the historical, political, social and cultural context of their lives, as opposed to knowledge about customs and rituals.

A shared language was viewed as intricately linked to culture and ethnicity, considered crucial for accurate knowledge and communication about needs and behavioural norms. One participant stated that:

*We have the words in our language, but they might not be translatable. They might come out harsher or softer than they're trying to portray. So you...can't use word for word in most cases...you'd have alternatives and it might not be as it's supposed to come out or exactly what the person is feeling*. [Patel, 2016]

In response to this need, services often did not have interpreters arranged or provided. This led to professionals making assumptions about their English proficiency, misunderstanding them and often missing information. This created distress and alienation for service users, preventing them from fully expressing themselves and relying on support or advocacy from others. Some arranged for family members to accompany them, albeit reluctantly as “it isn’t right that you have to bring someone” [28]. This also inhibited them from speaking out about distress that may have involved the family.

Another important cultural aspect was their conceptualisation of emotional distress. Service users largely positioned distress as related to social disadvantage and stressful situations. Some believed that their distress was a normal reaction to their situation, suggesting that they were not “ill” or in need of coping strategies [52]. One service user emphasised the differences between Eastern and Western conceptions of distress:

*It's old fashioned...to think that physical and mental are different. Because what is happening physically in your body is affecting your mind, so no they are not two separate things...I think doctors need to be mindful of that. Western medicine needs to change, accepting that all the things are linked*. [Patel, 2016]

These differences led to concerns about medication as a first-line treatment for emotional distress, especially regarding the side effects and long-term impact: “it’s not good to take this medication for long…because it’s not good for you” [52]. Many therefore regarded medication as a last resort and viewed doctors as limited in their ability to effect real change, since “doctors only give medical help…they do not have knowledge of problems that worry people” [59]. Their role was seen as limited to signposting and prescribing as they only treated the biomedical symptoms and not the underlying social causes.

Service users further described the importance of faith in their conceptualisation of distress. This was a protective factor, conferring mental strength, motivation and comfort, which was central to their ability to manage difficulties. Faith influenced the attitudes around distress and help-seeking, as well as the use and experiences of statutory services. For example, advice from spiritual healers was viewed as more credible than that of professionals, and service users often sought spiritual support alongside medical treatment. Accounting for religious or spiritual factors was considered crucial and at times privileged over shared ethnicity with professionals.

However, religion in healthcare was experienced as “knowledge first and faith last” [Thompson, 2010]. Service users felt that these needs were rarely acknowledged and that they could only volunteer this information if professionals appeared to accept spiritual explanations themselves. This impacted on their engagement and rapport with professionals:

*It’s one thing to have knowledge about something but it’s another to live it right? My counsellor said he didn’t understand some of the things we did and why we did them – that worried me…after a while I stopped going*. [28]

## Discussion

The findings indicate that South Asian service users experienced multiple barriers to accessing mental health services: a lack of information and effective communication from services, stigma from within the Asian community and wider society, mistrust of White and Asian professionals, a pervasive lack of cultural sensitivity, inadequate interpreting support and limited collaboration in decision-making processes. Previous research supports these findings but tends to locate the barriers within the South Asian community by focusing on differences in awareness and cultural norms. Since increased knowledge of “explanatory models” has not led to improvements in service use or outcomes [3–5], it is argued that South Asian service users face challenges in asserting their candidacy for healthcare utilisation due to multiple barriers located *within services*. This raises concerns about appropriate access to more specialist services, given that satisfactory provision and utilisation of primary and community services is often a prerequisite for further engagement.

### Importance of Trust for South Asian Communities

The line of argument synthesis identified trust as a key factor influencing decisions to access or continue accessing mental healthcare. Service users were stuck between trusting White or Asian professionals, based on legitimate concerns regarding the subsequent validation, empathy and privacy each might provide. This raises questions about the utility of ethnic-matching, which proposes that matching the ethnicity of service users to professionals facilitates service delivery [60]. Ethnic-matching is usually approximate and can prevent exploration of other aspects of cultural identity that may be more relevant for service users, such as faith [61]. Despite this, research has found that service users from BAME communities had a positive preference for and perception of therapists of their own ethnicity [62]. Since fears of confidentiality being breached can marginalise service users from their families or communities, it is essential that this is explained in ways that can be understood [5]. Based on the review findings, what appeared important for South Asian service users was the use of a sensitive approach involving a commitment to confidentiality, a humble appreciation of their unique ethnic and cultural identity and a genuine interest to attend to their conceptualisation of distress and faith.

The reported mistrust of White professionals reflects the concept of “cultural mistrust” [63]. This posits that Black groups have developed a mistrust of White service providers as a result of their historical and contemporary experiences with racism and oppression [64, 65]. The service users in this review similarly expressed “healthy cultural suspicions” that their “distress may be misunderstood, misconstrued and/or pathologised, and their own beliefs severely compromised or at worst undermined” [25]. For South Asian communities, this may stem from the British colonisation of the Indian subcontinent and resulting partition which led to tragic and widespread poverty, loss and powerlessness, yet continues to be overlooked and silenced even today [66, 67]. Coupled with ongoing experiences of racism and discrimination in the UK [11, 12], this makes it difficult for South Asian service users to trust services which may represent the power and authority that was instrumental in the oppression of their families and continues to cause harm to their communities.

### The Powerlessness and Threat Experienced When Seeking Help

When service users took the risk and accessed services, they reported professionals sharing little to no information with them and exerting control over decision-making processes. Whilst these experiences are not limited to South Asian service users [68], they faced additional barriers to collaborative care. They reported interpreters not being arranged or provided, which has serious implications for the quality of healthcare received. Language differences can hinder awareness regarding how the healthcare system works and what it can and cannot provide [69], resulting in limited knowledge about rights and slower progression from assessment to intervention [28, 52, 55]. It can also decontextualize the meaning of their distress, contributing to misunderstanding and misinterpretation by healthcare professionals [5]. Alongside this, professionals frequently overlooked the importance of their faith. There is strong evidence that religious/spiritual beliefs have a positive impact on many aspects of physical and mental health, yet professionals often hesitate to enquire due to inadequate knowledge or fears of ethical violation [32].

These findings are validated by previous research, where doctors delivered less competent clinical performance to BAME groups due to presumptions about the intelligence and expectations of these groups [34]. Mental health professionals also held stereotypes regarding South Asian cultures as repressive and inferior to a Western cultural ideal and therefore pathologised the culture as predisposing towards emotional distress [35]. Coyle (1999) posits that threats of being stereotyped, disempowered and devalued can impact on service users’ sense of self, especially impacting on groups that are generally afforded less power in British society, such as women and people from BAME backgrounds [68]. Given the traumatic history of colonial oppression and racial discrimination inherent in the lived experience of South Asian groups, service users were understandably reluctant to receive care from professionals who threatened and diminished their autonomy, values and understanding of distress. Their traumatic experiences of help-seeking further confirmed their mistrust of services.

### Distanced from Services Through Cultural Dissonance

The review therefore supports the suggestion that cultural dissonance generates low permeability; South Asian service users construed and presented their emotional distress differently from the imagined “ideal” user of mental health services, leading to a dissonance between their expectations from services and the intended use of services [27]. South Asian service users placed importance on the family and wider community and preferred a holistic conceptualisation of distress that accounted for their cultural traditions, social circumstances and faith. Whilst this reflects Eastern traditions that place value on balance, social integration and collective harmony, the Western “ideal” values self-sufficiency, personal autonomy and efficiency. This was evidenced by the emphasis on biomedical treatment options and individualistic models of “recovery” [5], which alienated South Asian service users and prolonged the threat to their cultural identity.

Over recent years, alternative options such as mindfulness and yoga have grown within mainstream healthcare, practices which are rooted in Eastern traditions and philosophy originating from the Indian subcontinent. However, the “whitewashing” of these traditional practices means that they often exclusively appeal to White middle-class groups who benefit from their secularised focus on increasing personal wellness and fitness [70, 71]. This appropriation of sacred ancestral traditions, alongside racist stereotypes of South Asian groups lacking “psychological mindedness” to engage with talking therapies [25, 35], may perpetuate cultural mistrust and marginalise South Asian service users from asserting their candidacy for nonmedical interventions that may align better with their values and needs. In response to this dissonance, some service users negated the applicability and relevance of mental healthcare, relying instead on self-determined coping mechanisms. This may be understood as means to preserve their cultural identity and protect themselves against the alienation and powerlessness experienced when using Eurocentric services [25].

### The Rigid Barrier of Eurocentricity

It has been suggested that improving the “mental health literacy” of South Asian communities could improve their awareness of services and attitudes towards help-seeking [1]. This approach assumes that adopting a Western cultural understanding of distress will lead to improved service use and outcomes for all cultural groups. Although this may alleviate barriers that are mainly cognitive or informational, determinants of help-seeking include social, interpersonal and affective factors [19]. The ongoing immigration of South Asian people to the UK also means that these communities are continually shifting in many heterogeneous characteristics, such as their generational status and levels of acculturation. Instead of providing a “recovery-enhancing environment that respects their beliefs, culture, faith, spiritual needs and values” [2], the findings suggest that healthcare professionals neglected these individual needs and inadvertently expected service users to adopt the Western norms and values through which services were delivered, maintaining their feelings of powerlessness and alienation.

Despite their longstanding dissatisfaction with statutory provision, South Asian service users may have no alternatives available locally and therefore risk their personal and cultural identity being threatened at times of crisis [60]. However, growing evidence points to the importance of understanding the social determinants of health over simplistic biological theories of “mental illness” and contextualising emotional distress as a “normal reaction to abnormal circumstances” [14, 31]. This would appear to be especially important for South Asian communities, who are disproportionately impacted by structural socioeconomic inequalities that contribute to psychological distress and can themselves pose barriers to accessing mental healthcare [13, 14, 29]. Their concerns regarding the overreliance on biomedical treatments are further validated by growing survivor-led campaigns and published literature regarding the harmful effects of psychiatric drugs [72–74].

This evidence challenges the notion that these groups are unintelligent or “inferior” in how they conceptualise emotional distress [34, 35] and raises serious concerns about the biomedical model which is largely held as legitimate and unproblematic by primary and community mental health services, but cannot do justice to the traumatic and holistic nature of their experiences [5, 60]. Together, these multiple barriers persist in “othering” South Asian communities and undermining their personal beliefs and traditional ways of understanding and addressing emotional distress and social suffering [75]. It has therefore been argued that it is not South Asian service users, but mental health services, that are “hard to reach”, given their lack of understanding, flexibility and communication due to stigma towards these groups, rigid Eurocentric practices and institutional racism [5, 32, 76].

### Considering Ways Forward

Although government policy mandated that all mental health practitioners develop their “cultural competence” through dedicated training in race equality [2], this has not yet led to marked changes in clinical practice and has been suggested to reproduce institutional racism by continuing to “other” marginalised communities [32]. Instead, it has been recommended that service providers establish “cultural safety” by addressing power imbalances inherent in the healthcare system and critically examining “how this influences usually unconscious assumptions and comparisons about others” [77, 78]. Since the definition and success of culturally safe practice is determined by the service user rather than the provider, the power of the interaction would be transferred to South Asian service users, empowering them to assert their candidacy and be actively involved in decisions about service delivery.

Where mainstream services have failed to engage South Asian service users, voluntary and community-based organisations have shown to be more effective, due to higher levels of trust, personalised support and being located in the communities they serve [76, 79]. For example, traditional healing practices and culturally adapted counselling/therapy are integrated with informal peer support and advocacy for social welfare, enabling a holistic approach to understanding and addressing emotional distress. However, these organisations rely on the enthusiasm and commitment of dedicated individuals and on increasingly limited funding and resources, so cannot mitigate the failures of statutory services [80]. It is therefore imperative that mainstream services work towards establishing cultural safety by improving their collaborative care approaches and cultural sensitivity and work alongside community-based organisations to facilitate trust and engagement with South Asian communities [60, 79, 80].

Community engagement can take many forms, including service user involvement, peer support or peer-delivered interventions and empowerment of the community to identify and take action to meet their own needs [81]. Consistent involvement of South Asian service users in the design of services has yet to be achieved across primary care trusts in the UK, despite being a key outcome of the Race Relations (Amendment) Act 2000 [2, 82, 83]. Similarly, peer support models remain in their infancy in the UK, generally limited to community-based or third-sector organisations, although they have been introduced into mainstream mental healthcare in other countries [84, 85]. These approaches could hold significant benefits for South Asian service users by offering a space for solidarity grounded in their lived experiences and social action against oppressive conditions and practices [32]. They could also increase the understanding of service providers and contribute to reducing the reported power imbalance. However, dedicated resources and support are essential for community engagement to lead to sustainable changes [76].

### Strengths and Limitations of the Review

This literature review is the first attempt to examine the help-seeking experiences of British South Asian service users in community mental healthcare through a comprehensive search strategy. This enabled a more transparent and reproducible process than using a search with purposive sampling, which would have been likely to omit some studies [39]. Analysing the research using the principles of meta-ethnography facilitated a detailed and deeper insight into the perceptions and experiences of service users. However, syntheses of this nature are distanced from the lived experiences of the participants they represent, since the studies themselves have interpreted the participants’ data [86]. Many of the studies also had smaller sample sizes, making a valid and reliable conclusion difficult. The quality and reporting of research in this area could be improved through researchers providing adequate information about the method and process of qualitative analysis and the reflexive steps taken to address potential biases. The dearth of recent published literature focusing on the specific experiences of South Asian service users is an important consideration for future research, alongside building the evidence on community-based and culturally sensitive models of mental healthcare, including assessment and intervention methods, that may be better placed to meet their needs.

It is acknowledged that there is a “category fallacy” when identifying South Asian people as a homogenous group since there is a wide range of religious, linguistic, cultural and economic diversity in migrants who originate from countries on the Indian subcontinent [87]. Also, since most studies included both first- and second-generation immigrants in their samples, it was difficult to focus on either generation separately without compromising on other valuable aspects of the data. Whilst this allowed for various perspectives to be considered, it may have obscured differences in help-seeking arising from acculturation [88]. Despite these limitations, this review is the first to investigate the views of South Asian service users separately from other ethnic groups and service providers, giving voice to their unique collective experience. Future studies should investigate the views of these groups separately in order to account for the diverse heterogeneity in experiences, for example, between specific ethnic or generational groups.

Several groups were excluded from this review whose experiences represent a gap in the literature and therefore warrant further research. Using age as an inclusion/exclusion criterion meant that the views of children and adolescents were not represented. Alongside this, the experiences of families and caregivers is also important, since they often influence decisions to seek professional support and can be involved in sharing information with both service users and providers. Furthermore, this review explored the lived experiences of “potential” and “actual” service users, based on the assumption that if barriers were understood and addressed, they would be willing and able to engage with mental health services. However, this excludes the perspective of people who choose not to access services intentionally despite being able to do so; understanding their views and experiences may help services to appreciate their choices around help-seeking.

### Implications for Policy and Clinical Practice

Much of the reported dissatisfaction has been expressed to service providers over the past 20 years; the failure to act and make lasting improvements has perpetuated the experience of powerlessness for South Asian communities. In order to address the identified barriers in accessing mental healthcare, services need to consider how their policies and practices effect underutilisation. As a minimum, they must provide service users with genuine options and choices that directly influence their quality of care: professional interpreters, information about their rights and prescribed medications, the inclusion of family members where appropriate and healthcare professionals that can attend to self-reported important aspects of their identity. This could begin to increase the permeability of community services, moderate the power imbalances experienced and enable South Asian service users to assert their needs and candidacy for more specialist services.

Service providers also need to consider the wider structural factors that influence the wellbeing of South Asian service users and stimulate the ongoing processes of “cultural humility” and antiracism. This requires healthcare professionals to engage in lifelong learning and critical self-reflection to dismantle assumed superiority over South Asian cultural norms and understand the impact of institutional racism and other systems of oppression on mental health. This could support professionals to cocreate a holistic conceptualisation of wellbeing that respects service users’ personal values and faith, maintains curiosity towards the unique cultural and sociopolitical context affecting their lives and thereby refrains from individualising or pathologising their distress. Such processes must be considered integral to establishing cultural safety and delivering quality mental healthcare for a diverse population and embedded through professional training programmes and sustained workforce development.

At an organisational level, there must be a commitment to strengthening trust and community engagement. This must be prioritised over simply promoting the availability of services; although the current findings support this requirement, it is argued that services must first cultivate cultural safety, so as not to continue reinforcing distance when services are utilised. Therefore, considering how information is best communicated and by whom is vital, keeping in mind the potential to perpetuate cultural mistrust through applying Eurocentric concepts of wellbeing and distress. Greater and consistent collaboration between statutory services and local community-based groups could facilitate continuous learning and effective decision-making through mutually respectful partnerships. This would support the growth of meaningful spaces for South Asian service users to define and evaluate the success of healthcare services and contribute to national policies for service planning and development. It could also empower them to take local action in their communities to meet their emotional and social needs. With sufficient funding and resources, more relevant models of healthcare could also be mobilised, such as peer support or peer-delivered interventions.

## Data Availability

The data included and analysed was obtained from the fifteen identified articles. Informed consent was obtained by the authors of these original studies so was not reproduced for this review. The annotated papers and analysis tables can be made available upon request.
